# CT-based assessment of bear-inflicted maxillofacial injuries: evaluation using the facial injury severity scale and its association with hospitalization duration

**DOI:** 10.1007/s11604-025-01827-0

**Published:** 2025-07-01

**Authors:** Kento Hatakeyama, Takahiro Otani, Kasumi Satoh, Hideomi Tsuchida, Tomoki Tozawa, Motoko Konno, Shinsuke Suzuki, Takafumi Tezuka, Shunji Mugikura, Akira Hayakawa, Takechiyo Yamada, Hajime Nakae, Naoko Mori

**Affiliations:** 1 Department of Radiology, Akita University Graduate School of Medicine, 1-1-1 Hondo, Akita, Akita 010-8543 Japan; 2 Department of Emergency and Critical Care Medicine, Akita University Graduate School of Medicine, 1-1-1 Hondo, Akita, Akita 010-8543 Japan; 3 Department of Otorhinolaryngology, Akita University Graduate School of Medicine, 1-1-1 Hondo, Akita, Akita 010-8543 Japan; 4 Department of Dermatology and Plastic and Reconstructive Surgery, Faculty of Medicine, Akita University Graduate School of Medicine, 1-1-1 Hondo, Akita, 010-8543 Japan; 5 Division of Image Statistics, Tohoku Medical Megabank Organization, Tohoku University, 2-1 Seiryo-Machi, Aoba-ku, Sendai, Miyagi 980-8573 Japan; 6 Department of Forensic Sciences, Akita University Graduate School of Medicine, 1-1-1 Hondo, Akita, Akita 010-8543 Japan

**Keywords:** Bear-inflicted injury, Maxillofacial injury, Facial injury severity scale

## Abstract

**Purpose:**

Bear-inflicted injuries are often associated with severe maxillofacial injury. This study aimed to evaluate CT findings of bear-inflicted injuries using the Facial Injury Severity Scale (FISS) and other trauma grading systems, with a particular focus on the relationship between FISS scores and hospitalization duration as well as the characteristics of maxillofacial injuries.

**Materials and methods:**

This retrospective study included 31 patients with bear-inflicted injuries who underwent whole-body CT. Maxillofacial injury severity was assessed using FISS, while head and thoracic injuries were evaluated using the Marshall Head-CT Classification and the AAST Chest Wall Injury Scale, respectively. Patients were categorized into short (≤ 18 days) and long (> 18 days) hospitalization groups based on the median hospitalization duration. Univariate and multivariate analyses were performed to identify factors associated with hospitalization duration.

**Results:**

Patients in the long hospitalization group had higher FISS scores (p = 0.01) and AAST chest wall injury grades (p = 0.03), with no significant differences in Marshall Head-CT Classification. Multivariate analysis confirmed that both FISS scores and AAST chest wall injury grade were independently associated with hospitalization duration. A detailed analysis of FISS scores revealed significant bilateral correlations between right and left midface scores (p < 0.01) and right and left upper face scores (p < 0.01). Additionally, significant correlations were observed between midface and upper face scores.

**Conclusion:**

FISS scores and AAST chest wall injury grades were significantly associated with hospitalization duration in bear-inflicted injuries. The midface and upper face injuries often occur simultaneously and bilaterally, possibly due to bears targeting the upper face to weaken their opponent.

## Introduction

Recent studies indicate an increasing incidence of bear-inflicted injuries, with attacks traditionally occurring in remote rural areas and now increasingly reported within human living spaces [1]. These attacks predominantly target the face, with 90% of victims affected by maxillofacial injuries, including fractures, extensive bleeding, and skin detachment [2, 3]. Severe bear-inflicted injuries require early transportation to a tertiary medical institution, emergency blood transfusions, intubation, and surgical intervention for complex injuries [3, 4]. Delays in rescue and medical treatment can significantly impact patient outcomes [4].

Computed tomography (CT) is essential for diagnosing and evaluating the bear-inflicted injuries. CT reveals complex maxillofacial fractures, displaced bone fragments, and associated soft tissue injuries [5, 6]. The comminuted fractures of the zygomatic complex, orbital floor, and maxilla are frequently observed [5, 7]. Eye injuries occur in 59% of cases, and 23.5% of patients are permanently blind in one eye [8]. Early CT scans are necessary for surgical planning of staged reconstruction and outcome prediction, as they allow for the assessment of fracture patterns and displacement and identification of critical structure involvement, including maxillofacial injuries, as well as head and trunk injuries [5, 7].

The Facial Injury Severity Scale (FISS) has been proposed as a standardized tool for assessing maxillofacial injury severity and predicting patient outcomes [9]. The FISS indeed incorporates anatomical regions in its scoring system, dividing maxillofacial injuries into three distinct zones: the upper face, midface, and mandible (lower face). Each anatomical region is assigned specific scores based on the type and complexity of fractures. Upper face fractures, including those of the frontal bone and frontal sinus, typically receive higher scores due to their proximity to the brain as critical structures. Higher FISS scores indicate more severe injury and more extended recovery periods, with scores above 11 often necessitating treatment across multiple regions [10, 11]. A significant correlation between FISS scores and hospitalization duration has been reported [10]. To the best of our knowledge, no studies have applied FISS to maxillofacial injuries caused by bears.

This study aims to evaluate the CT findings of bear-inflicted injuries using FISS along with another grading system for head and body injuries and to investigate the relationship between FISS scores and hospitalization duration. Additionally, we performed a detailed analysis of the FISS scores to clarify the characteristics of bear-inflicted maxillofacial injuries.

## Materials and methods

### Patients

**Fig. 1:**
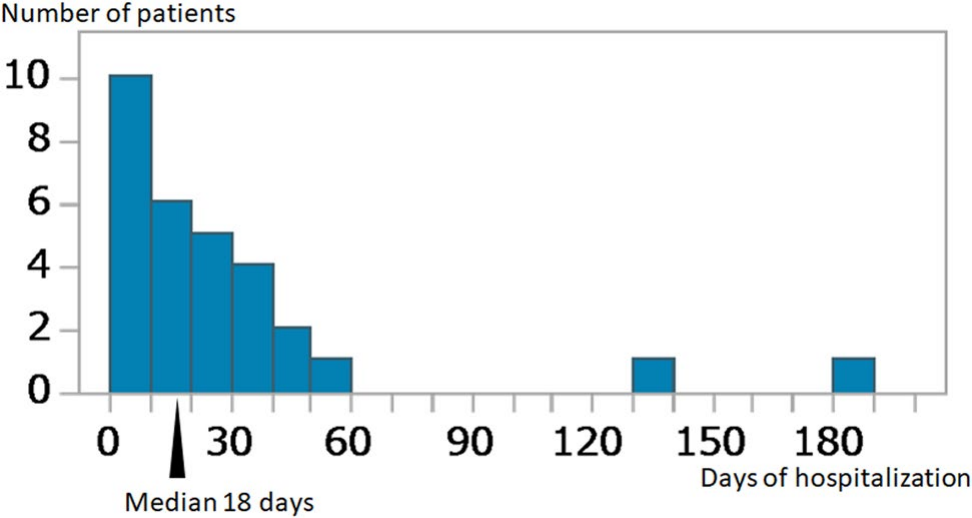
Histogram depicting the distribution of hospitalization duration among surviving patients (n = 30). The x-axis represents the number of hospitalization days, while the y-axis shows the number of patients. The median hospitalization duration was 18 days (arrowhead), with a range of 0–185 days

This retrospective study was approved by the institutional review board, with a waiver of the requirement for informed consent. The inclusion criteria were patients who underwent pan scan CT, including head, neck, chest, abdomen, and pelvis, for the evaluation of bear-inflicted injuries. To identify eligible cases, we conducted a search of our radiology reporting system and hospital information system from September 2005 to June 2024 for reports containing the terms “bear-inflicted injuries”. During this period, 31 patients with bear-inflicted injuries were included in the present study. Patient characteristics, including age, gender, and hospitalization duration, were obtained from the hospital information system. Hospitalization duration was calculated in days, and the median was determined using a histogram analysis. Patients with a hospitalization duration at or below the median were classified as the short hospitalization group, while those with a duration exceeding the median were classified as the long hospitalization group (Fig. 1). Death cases during hospitalization were excluded from the analysis.

### Image acquisition and assessment

All patients were scanned using a Revolution CT scanner (GE Healthcare Japan, Tokyo, Japan). For head and neck CT, images were acquired at a tube voltage of 120 kVp, with raw data obtained at a slice thickness of 0.625 mm and reconstructed at 2.5 mm. For whole-body CT, images were reconstructed at a slice thickness of 5 mm, covering the neck, chest, abdomen, and pelvis.

**Fig. 2:**
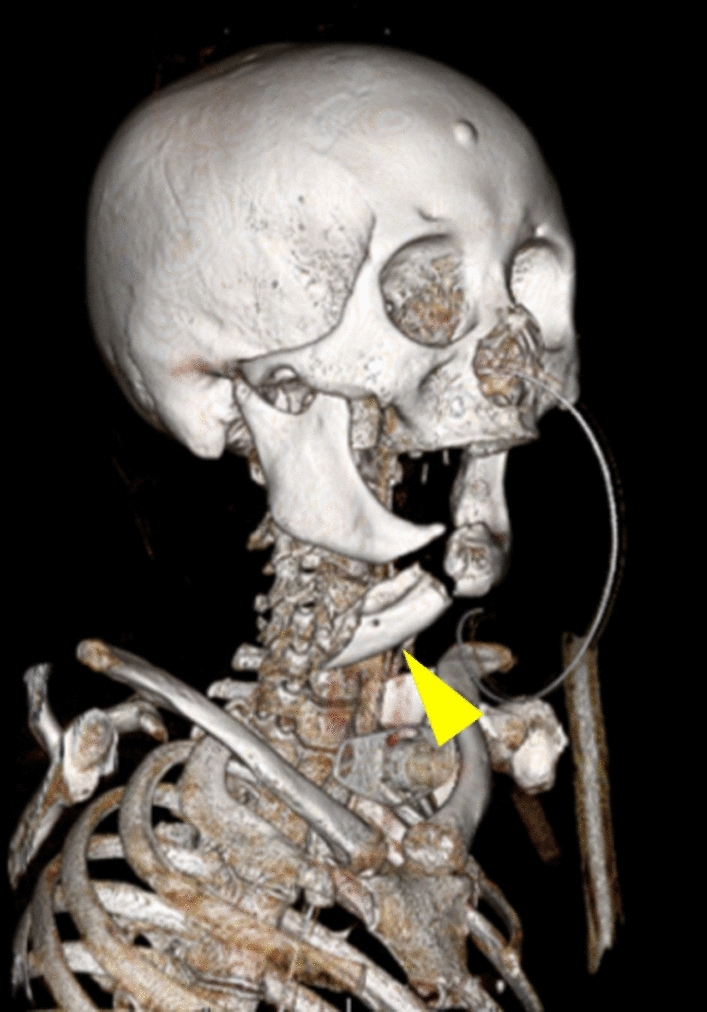
Three-dimensional volume-rendered CT reconstruction illustrating mandibular fractures in a 76-year-old female patient. The image shows fractures of the right mandibular body and symphysis, with caudal displacement of the bone fragments (yellow arrowhead)

**Fig. 3:**
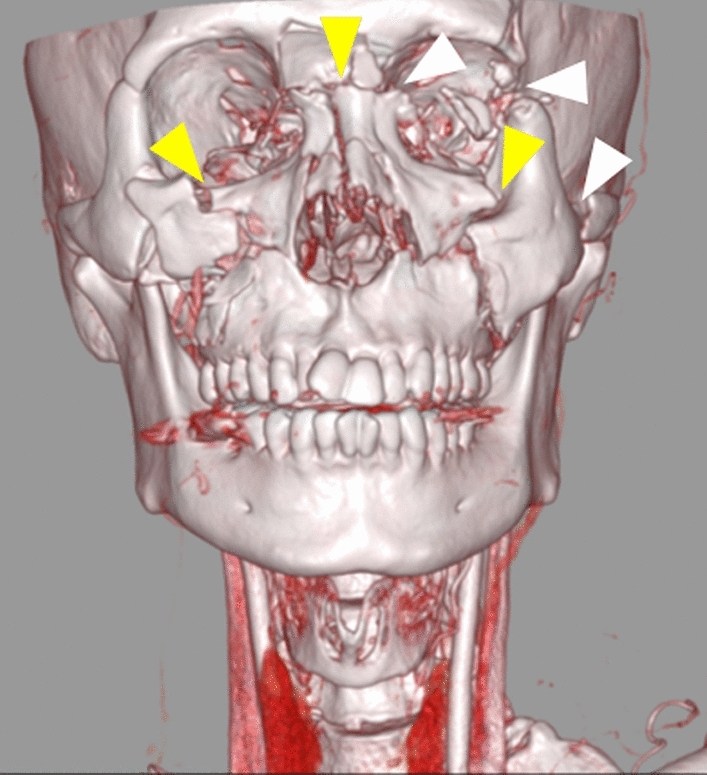
Three-dimensional volume-rendered CT reconstruction showing midface fractures in a 46-year-old male patient. The image illustrates bilateral Le Fort II fractures (yellow arrowheads; 2 points per side) and a left Le Fort III fractures with craniofacial separation (white arrowheads; 3 points per side)

**Fig. 4:**
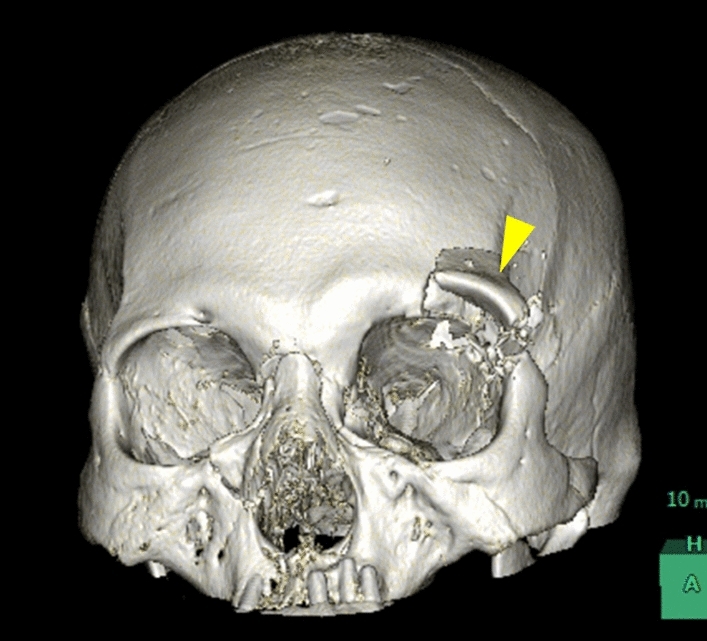
Three-dimensional volume-rendered CT reconstruction showing an upper face fracture in an 83-year-old female patient. The image illustrates a left orbital roof/rim fracture (yellow arrowhead; 1 point)

The severity of maxillofacial injury was independently assessed by two board-certified radiologists (with 16 and 20 years of experience in radiology, respectively, T.O. and N.M.) on the head and neck CT using the FISS with bilateral scoring [9]. Each fracture was assigned a numeric value, with separate scores recorded for the left and right sides where applicable. Mandibular fractures were classified as dentoalveolar, body, ramus, condyle, and coronoid (Fig. 2). The mandibular symphysis, being a midline structure, was assigned a single score without lateral differentiation (Table 1). In the midface, the dentoalveolar fracture, Le Fort fractures, Naso-orbital-ethmoid (NOE) fracture, zygomaticomaxillary complex (ZMC) fractures, and nasal fractures were scored bilaterally (Fig. 3). In the upper face, orbital roof/rim fractures and frontal sinus/bone fractures, including displaced fractures, and non-displaced fractures, were recorded separately for each side (Fig. 4). Soft tissue injuries were assessed separately for each side based on the total length of facial lacerations exceeding 10 cm (Table 1). The mandible, midface, and upper face regions were evaluated separately on the right and left sides, and the corresponding scores were calculated as the Right Mandible Score, Left Mandible Score, Right Midface Score, Left Midface Score, Right Upper Face Score, and Left Upper Face Score. Injuries were scored bilaterally, and the total scores for each side were calculated as the Right and Left FISS scores.

**Table 1:** Facial injury severity scale (FISS) with bilateral scoring

Region	Fracture type	Left	Right
Mandible	Dentoalveolar	1	1
	Body	2	2
	Ramus	2	2
	Symphysis	2	
	Condyle	1	1
	Coronoid	1	1
Midface	Dentoalveolar	1	1
	Le Fort I	1	1
	Le Fort II	2	2
	Le Fort III	3	3
	Naso-Orbital Ethmoid (NOE)	3	3
	Zygomatico Maxillary Complex (ZMC)	1	1
	Nasal	1	1
Upper face	Orbital roof/rim	1	1
	Displaced frontal sinus/bone fractures	5	5
	Non-displaced fractures	1	1
Facial laceration	Over 10 cm long	1	1

The same two radiologists assessed intracranial findings using the Marshall Head CT Classification, which categorizes traumatic brain injuries into six categories based on CT characteristics such as lesion type and degree of midline shift [12]. Diffuse Injury I indicates no visible intracranial pathology. Diffuse Injury II is defined by the presence of cisterns, a midline shift of 0 to 5 mm, and the absence of high- or mixed-density lesions larger than 25 cm^3^. Diffuse Injury III involves compressed or absent cisterns, with a midline shift of 0 to 5 mm, and no lesion exceeding 25 cm^3^. Diffuse Injury IV is characterized by a midline shift greater than 5 mm, without a lesion larger than 25 cm^3^. Evacuated Mass Lesion (Category V) refers to any lesion that has been surgically evacuated, while Non-evacuated Mass Lesion (Category VI) refers to a high- or mixed-density lesion larger than 25 cm^3^ that has not been surgically removed. In our 31 cases of bear-inflicted injury, cases with head injuries exhibited either subdural hematoma or cerebral contusion, each with a volume less than 25 cm^3^, which fall within the criteria for Diffuse Injury II in the Marshall Head CT Classification, a system consisting of six categories based on CT findings. Therefore, we evaluated the absence or presence of findings corresponding to Diffuse Injury II. In this study, chest, abdomen, and pelvis CT scans identified only thoracic injuries, which were evaluated using the AAST Chest Wall Injury Scale, a system with five severity grades. No abdominal or pelvic injuries were observed in any of the cases. According to this scale, Grade I injuries include contusions of any size, lacerations limited to the skin and subcutaneous tissue, and fractures that consist of either fewer than three closed, non-displaced rib fractures or a non-displaced clavicle fracture. In contrast, Grade II injuries encompass lacerations that involve the skin, subcutaneous tissue, and muscle, as well as fractures defined by three or more contiguous closed rib fractures, open or displaced clavicle fractures, non-displaced sternal fractures, or scapula fractures, whether open or closed [13]. Since only Grade II injuries were observed in this study, the AAST Chest Wall Injury Scale was used to assess only the absence or presence (Grade II) of injuries. Additionally, the radiologist assessed superficial and muscular injuries of the limbs and trunk using CT, identifying soft tissue contusions, lacerations, or muscular injuries without deeper structural involvement.

### Statistical analysis

For the primary analysis comparing the short and long hospitalization groups, 30 cases were analyzed, excluding the single fatal case. The inter-rater reliability of FISS scores between the two radiologists was assessed using intraclass correlation coefficients (ICC). The agreement regarding the presence or absence of findings in the Marshall Head CT Classification, the AAST Chest Wall Injury Scale, and superficial and muscular injuries of the limbs and trunk was assessed using κ statistics. Gender, the underlying medical conditions, the presence or absence of Diffuse Injury II of Marshall Head-CT Classification, the presence or absence of Grade II of AAST Chest Wall Injury Scale, and the presence or absence of superficial and muscular injuries of the limbs and trunk were compared between the short and long hospitalization groups using the chi-square test. Age and FISS scores were compared between these groups using the Mann–Whitney U test. To identify factors associated with short and long hospitalizations, we conducted a multivariate analysis using the two CT findings that had the lowest p-values in the univariate analysis and were considered likely to influence hospitalization duration. For the sub-analysis, all 31 cases, including the fatal case, were analyzed. As part of this sub-analysis, FISS scores for the mandible, midface, and upper face scores were summed separately for the right and left sides to obtain the right mandible score, left mandible score, right midface score, left midface score, right upper face score, and left upper face score. The relationships among these scores were examined using Pearson’s correlation coefficient. As a further sub-analysis of the FISS score, the scores for the right and left sides were summed (excluding the symphysis of the mandible as it is a midline structure), and the right and left FISS scores were compared using a paired t-test. All statistical analyses were conducted using JMP Pro 17 (SAS Institute Inc., Cary, NC, USA). A p-value < 0.05 was considered statistically significant.

## Results

**Fig. 5:**
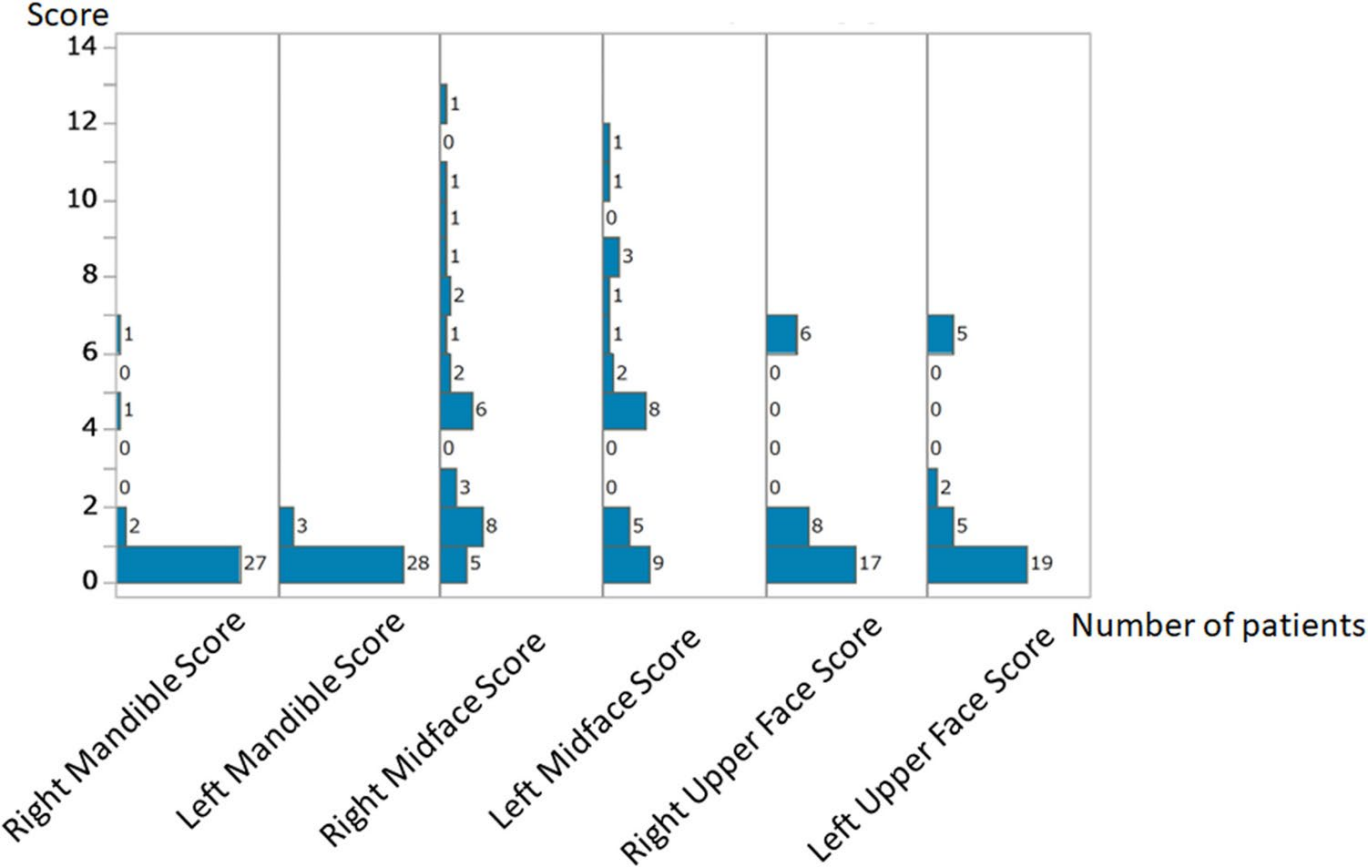
Histogram showing the distribution of Facial Injury Severity Score (FISS) components. The y-axis represents the FISS components scores, while the x-axis represents the number of patients. The Right and Left Mandible Scores were predominantly zero, with a few cases having nonzero values. The Right and Left Midface Scores exhibited a wider range, with some patients showing high scores. The Right and Left Upper Face Scores also varied, though a significant proportion of patients had low scores

**Fig. 6:**
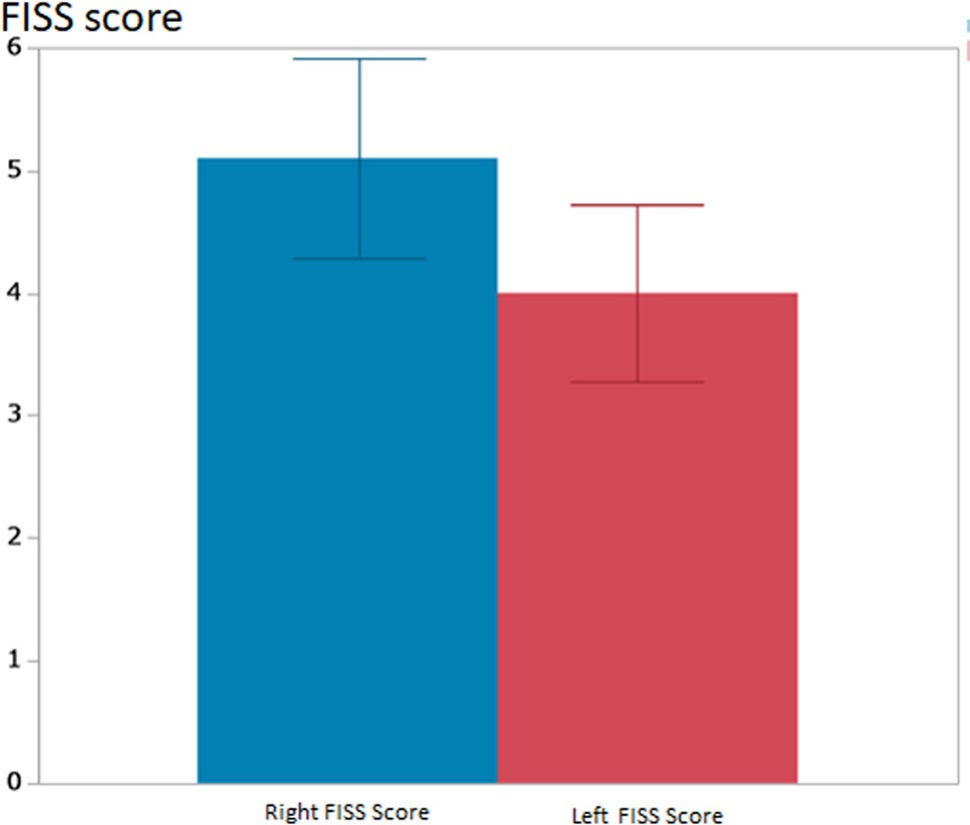
Comparison of Right and Left Facial Injury Severity Score (FISS). The Right FISS Score (blue) had a mean of 5.1, which was higher than the Left FISS Score (red) (mean: 4.0). However, a paired t-test showed no significant difference (P = 0.13). Error bars represent standard error

A total of 31 patients were included in the study. Of these, 9 (29.0%) were female and 22 (71.0%) were male. The median age was 72 years (range: 35–84 years). Among surviving patients (n = 30), the median hospitalization duration was 18 days (range: 0–185 days) (Fig. 1). The single patient who did not survive (n = 1, 3.2%) had a hospitalization duration of 3 days (Table 2). Patients were categorized into two groups based on hospitalization duration: the short hospitalization group (≤ 18 days) and the long hospitalization group (> 18 days) (Fig. 1). The ICC for FISS scores was 0.989 (95% CI 0.977–0.995), indicating almost perfect agreement. The κ values for the Marshall Head CT Classification, AAST Chest Wall Injury Scale, and superficial and muscular injuries of the limbs and trunk were all 1.0, indicating perfect agreement. In the univariate analysis, the FISS score was significantly higher in the long hospitalization group (13.8 ± 9.2) than in the short hospitalization group (6.2 ± 7.0) (p = 0.01) (Table 3). AAST chest wall injury grade was significantly higher in the long hospitalization group than in the short hospitalization group (p = 0.03). Gender, age, underlying medical conditions, Marshall Head-CT Classification, and Superficial and Muscular Injuries of the Limbs and Trunk showed no significant differences between the two groups. We selected the FISS score and AAST chest wall injury grade for multivariate analysis. The multivariate analysis confirmed that FISS score and AAST chest wall injury grade were independently associated with the differentiation between short and long hospitalization groups (*p* = 0.01 and p = 0.03) (Table 3). Regarding the FISS score distribution, the Right Mandible Score and Left Mandible Score were predominantly zero, with a few cases having nonzero values. The Right and Left Midface Scores showed a wider range of values, with some patients exhibiting higher scores. The Right and Left Upper Face scores also varied, though a significant proportion of patients had low scores (Fig. 5). Statistically significant correlations were observed between the Right and Left Midface Scores (*p* < 0.01) and the Right and Left Upper Face Scores (*p* < 0.01), indicating a bilateral relationship in these regions (Table 4). A significant correlation was also found between Right Upper Face Score and Right Midface Score (*p* < 0.01), Right Upper Face Score and Left Midface Score (*p* < 0.01), Left Upper Face Score and Right Midface Score (p = 0.03), Left Upper Face Score and Left Midface Score (*p* < 0.01) (Table 4). In contrast, the Right and Left Mandible Scores showed no significant correlation (Table 4). The Right FISS Score had a mean of 5.1 with a standard deviation of 4.5, which was higher than the Left FISS Score (mean: 4.0, standard deviation: 4.0); however, a paired test showed no significant difference (P = 0.13) (Fig. 6).

**Table 2:** Background factors of patients with bear-inflicted injuries

Variables (n = 31)	Values
Gender (Females/Males) (n (%))	9 (29.0)/22 (71.0)
Age (Median (range))	72 (35–84)
Days of hospitalization (Median (range))	
Alive (n = 30, 96.8%)	18 (0–185)
Death (n = 1, 3.2%)	3

**Table 3:** Univariate and multivariate analysis of background factors and CT findings between short and long hospitalization groups

	Short hospitalization group (n = 15)	Long hospitalization group (n = 15)	***p*** value (univariate)	***p*** value (multivariate)
Gender (females/males)	4 (26.7)/11 (73.3)	5 (33.3)/10 (66.7)	1.00	NA
Age (mean ± SD)	67.4 ± 12.9	69.3 ± 15.4	0.44	NA
Underlying medical conditions (absence or presence)				
Diabetes	12 (80.0)/3 (20.0)	14 (93.3)/1 (6.67)	0.60	NA
Hypertension	12 (80.0)/3 (20.0)	14 (93.3)/1 (6.67)	0.60	NA
Thyroid disorder	14 (93.3)/1 (6.67)	15 (100)/0 (0)	1.00	NA
Dementia	15 (100)/0 (0)	13 (86.7)/2 (13.3)	0.48	NA
CT findings				
FISS	6.2 ± 7.0	13.8 ± 9.2	0.01*	0.01*
Marshall head-CT classification (absence or presence of diffuse injury II)	12 (80.0)/3 (20.0)	14 (93.3)/1 (6.7)	0.33	NA
AAST chest wall injury grade (0/2) (absence or presence of grade 2 injury)	15 (100.0)/0 (0.0)	12 (80.0)/3 (15.0)	0.03*	0.03*
Superficial and muscular injuries of the limbs and trunk	4 (26.7)/11(73.4)	8 (53.3)/7 (46.7)	0.26	NA

Short hospitalization group: hospitalization duration≦18 days

Long hospitalization group: hospitalization duration > 18 day

*SD* standard deviation, *FISS* facial injury severity scale, *NA* not applicable

**Table 4:** *P* values of Pearson’s correlation between Facial Injury Severity Score (FISS) components

	Right mandible score	Left mandible score	Right midface score	Left midface score	Right upper face score	Left upper face score
Right mandible score	–	–	–	–	–	–
Left mandible score	0.99	–	–	–	–	–
Right midface score	0.52	0.43	–	–	–	–
Left midface score	0.42	0.45	< 0.01*	–	–	–
Right upper face score	0.58	0.27	< 0.01*	< 0.01*	–	–
Left upper face score	0.45	0.30	0.03*	< 0.01*	< 0.01*	–

–: not applicable

* indicates statistical significance

**Fig. 7:**
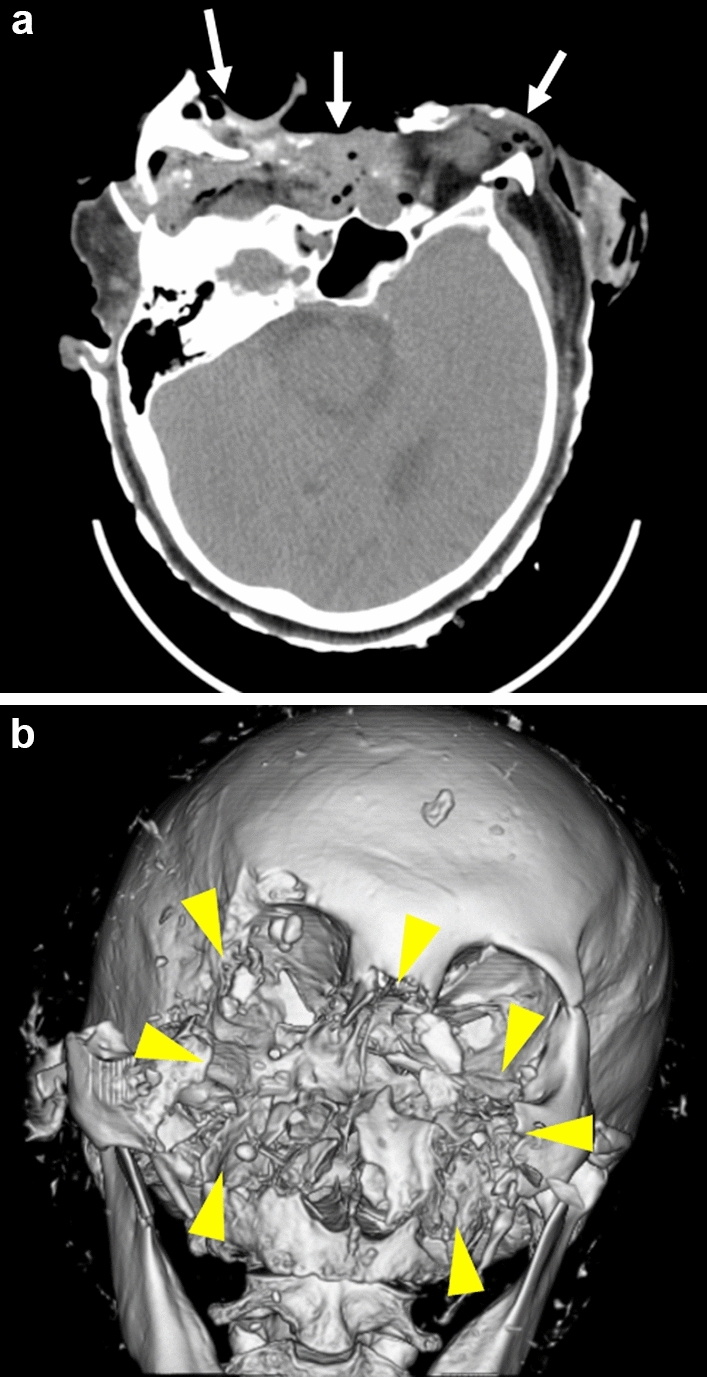
CT images of an 84-year-old male patient with severe bilateral midface and upper face fractures resulting in a Facial Injury Severity Score (FISS) of 31. **a** Axial CT scan demonstrating extensive facial fractures (white arrows), **b** Three-dimensional volume-rendered CT reconstruction illustrating the severity of bilateral midface and upper face fractures (yellow arrowheads). The patient underwent irrigation and debridement for facial trauma and died of septic shock three days later

Among the 31 cases of bear-inflicted injury, one fatal case had a FISS score of 31 and a right subdural hematoma, classified as Diffuse Injury II according to the Marshall Head CT Classification. However, the case had an AAST Chest Wall Injury Scale grade of 0, with the absence of superficial and muscular injuries of the limbs and trunk (Fig. 7). The patient underwent irrigation and debridement for facial trauma and died of septic shock three days later.

## Discussion

This study evaluated the CT findings of bear-inflicted injuries using the FISS, Marshall Head-CT Classification, AAST chest wall injury grade, and Superficial and Muscular Injuries of the Limbs and Trunk. The FISS score and AAST chest wall injury grade were significantly associated with hospitalization duration. Additionally, the inter-observer agreement for the FISS scores, Marshall Head CT Classification, AAST Chest Wall Injury Scale, and superficial and muscular injuries of the limbs and trunk was excellent, supporting the reliability of our imaging evaluations.

In our study, 71.0% of bear attack victims were male, and the median age was 72 years, indicating that the affected population was predominantly elderly. Previous reports have documented that bear attack victims are overwhelmingly male and almost exclusively adults, which is consistent with our findings [14, 15]. The mean hospitalization duration has been reported as 22 days [16], which is not markedly different from the median of 18 days in our study. Fatalities due to bear-inflicted injuries were observed in one case (3.2%) in our study. Bear attacks of the present study in Akita Prefecture, located in the northeast region of Japan, are believed to be caused by the Asiatic black bear [3, 17]. A previous study analyzing 417 cases of bear-inflicted injuries attributed to black bears reported a mortality rate of 2.4% [18], which is comparable to our findings. However, our mortality rate of 3.2% reflects only cases of bear-inflicted injuries among patients transported to the hospital. Cases of prehospital deaths, including those discovered as unexplained deaths and subsequently examined by forensic autopsy, were not included in our analysis, which may affect the accuracy of the mortality rate.

Our findings demonstrate that patients in the long hospitalization group had significantly higher FISS scores and higher AAST chest wall injury grades than those in the short hospitalization group in multivariate analysis. This result is consistent with previous studies on maxillofacial trauma, including those not limited to bear-inflicted injuries, where higher FISS scores have been associated with increased treatment complexity, longer recovery times, and the need for multidisciplinary care [9, 11]. In a previous study, FISS scores ranged from 1 to 12 [19]. In contrast, the FISS scores in our study, which focused on bear-inflicted injuries, showed a wider distribution, with a maximum score of 33. This finding suggests that bear- inflicted maxillofacial injury tends to be more severe than general maxillofacial injuries. Additionally, the Marshall Head-CT Classification (i.e., the presence or absence of Diffuse Injury II) did not differ significantly between the short and long hospitalization groups in univariate analysis. In our cohort, head injuries consisted of one case of cerebral contusion and four cases of subdural hematoma. These findings indicate that, in bear-inflicted injury cases, maxillofacial injury and chest injury might have a greater impact on hospitalization duration than head injuries.

The distribution of maxillofacial injuries in our study showed that midface scores were generally high. This finding aligns with previous reports indicating that maxillofacial fractures caused by bear attacks predominantly occur in the midface region [20]. Previous reports concerning laterality in bear-inflicted injuries include both unilateral and bilateral cases. Unilateral cases have been associated with severe maxillofacial injury and eye injuries, with one study reporting that 15% of cases had unilateral eye injuries, all resulting in vision loss [21]. In contrast, bilateral maxillofacial fractures have also been reported, although these findings are based on a limited number of cases, and no large-scale statistical study has conclusively demonstrated clear laterality differences in bear-inflicted injuries [22–24]. In our sub-analysis, we observed statistically significant bilateral correlations in midface and upper face injuries, with a corresponding correlation between midface and upper face scores. Conversely, mandibular fractures appeared to occur independently on each side, possibly reflecting asymmetric bite forces or unilateral defensive injuries sustained during an attack. Furthermore, no significant differences were found when comparing the FISS scores between the right and left sides. Based on these results, we demonstrated that in bear-related injuries, midface and upper face injuries often occur simultaneously and are frequently bilateral. The frequent bilateral injuries to the midface and upper face may be attributed to the fact that bears, as highly intelligent animals, tend to target the upper part of the face to weaken their opponent and prevent retaliation [25]. In a previous study of 469 patients with maxillofacial trauma, mandibular fractures were the most common injury type [11]. The study population mainly consisted of patients who had sustained injuries from traffic accidents and interpersonal violence. Both of these causes are typically associated with mandibular trauma. In contrast, our findings suggest that midface fractures are more characteristic of bear-inflicted injuries. A previous study reported that eye injuries occur in 59% of cases, and 23.5% of patients are permanently blind in one eye [8]. Although eye injuries are not included as an evaluation item in the FISS scoring system, they are believed to significantly impact post-injury aesthetics and quality of life (QOL). It has been reported that even after the acute phase and hospital discharge, patients with maxillofacial injury experience an increased incidence of Post-Traumatic Stress Disorder (PTSD), higher rates of alcohol dependence, and a decline in QOL [26]. Therefore, if outcomes such as appearance and QOL are to be assessed, a separate scoring system that includes eye injuries may be necessary.

Although no simplified version of the FISS has been established to date, we explored the potential utility of region-specific scores in the supplemental analysis. In Table 5, we compared the FISS sub scores between the short and long hospitalization groups. Notably, the Total Midface Score showed a statistically significant difference between the two groups, with a p-value comparable to that of the Total FISS Score. This finding suggests that the Midface Score alone may potentially serve as a simplified indicator of facial injury severity. However, due to the limited sample size in each group (n = 15), the generalizability of this finding remains uncertain. Further research with larger patient cohorts is warranted to validate this approach and to develop a simplified assessment method for clinical use.

In this study, the one mortality case was an 84-year-old man with a FISS score of 31 and a right subdural hematoma. Previous studies have not reported a direct association between FISS scores and mortality. Instead, factors such as age, Glasgow Coma Scale (GCS) scores, and the Injury Severity Score (ISS) have been identified as being associated with mortality in maxillofacial injury [27]. The ISS calculates trauma severity by summing the squares of the three most severe injuries, each scored from 1 (minor) to 6 (unsurvivable), based on the Abbreviated Injury Scale (AIS) [28]. The AIS is a standardized system that assigns severity scores to individual injuries across different body regions, providing the foundation for ISS calculations. Since there was only one fatality in this study, further investigation into factors related to mortality in bear-inflicted injuries would require multi-institutional studies or the inclusion of fatal cases from forensic analyses.

AIS is a widely used scoring system for assessing trauma severity. However, AIS evaluation, which incorporates multiple aspects such as clinical findings, surgical reports, and imaging data and relies on detailed coding, is time-consuming. In contrast, CT-based scoring systems, such as FISS, the Marshall Head-CT Classification, and the AAST Chest Wall Injury Scale, allow for an objective assessment of bear-inflicted injury severity based solely on radiological findings. The CT-based scoring in this study was a combination of existing scoring systems proposed for different anatomical regions. Although AIS was not used in this study, a key strength of our study is that FISS and the AAST Chest Wall Injury Scale, both derived from objectively obtained initial CT images, were found to be associated with hospitalization duration.

This study has several limitations. First, the sample size was small due to the rarity of bear-inflicted injuries. Larger, multi-institutional studies are needed to validate our findings. Second, this study was retrospective and compared initial CT findings from bear-inflicted injuries with hospitalization duration without evaluating treatment variability or post-discharge QOL. Future studies should investigate the relationship between initial CT findings, treatment variability, and post-discharge QOL to provide a more comprehensive understanding of outcomes in bear-inflicted injuries. Moreover, potential postoperative complications, such as infection or occlusal dysfunction, were not assessed in this study. These factors may directly influence not only long-term outcomes and patient QOL, but also hospitalization duration itself. Therefore, future studies should investigate the relationship between initial CT findings, postoperative complications, treatment variability, and post-discharge QOL to provide a more comprehensive understanding of outcomes in bear-inflicted injuries.

In conclusion, FISS score and AAST chest wall injury were significantly associated with hospitalization duration in bear-inflicted injury. In a detailed analysis of FISS scores, midface, and upper face injuries often occur simultaneously and bilaterally.

## Appendix

See Table 5.

**Table 5:** Univariate analysis of total FISS score and the detailed scores for each facial region between short and long hospitalization groups

	Short hospitalization group (n = 15)	Long hospitalization group (n = 15)	***p*** value (univariate)
Total FISS score	6.2 ± 7.0	13.8 ± 9.2	0.01*
Total mandible score	0	0.9 ± 1.8	0.02*
Total midface score	3.9 ± 4.1	8.9 ± 5.9	0.01*
Total upper face score	1.9 ± 3.6	3.1 ± 4.4	0.34
Total facial laceration score	0.4 ± 0.6	0.9 ± 0.5	0.02*

Short hospitalization group: hospitalization duration≦18 days

Long hospitalization group: hospitalization duration > 18 day

*FISS* facial injury severity scale
